# Raising Money for Hospitals

**Published:** 1922-12

**Authors:** 


### RAISING MONEY FOR HOSPITALS.

At the present time almost every charitable institution in this country, with the exception of the fortunate few which are substantially endowed, is faced with the problem of financing its work. Methods of all kinds are being tried, ranging from the ambitious Hospitals of London Combined Appeal to the humble flag-day in the country village for the county hospital.

There is not sufficient evidence at present either to criticise or to praise the London campaign. We do not yet know the eventual cost, what commissions have been paid or what is the final result; nor from the point of view of the individual hospital, struggling from year to year to raise money, is it of special advantage to consider the London hospital propaganda scheme. There is only one Prince of Wales whose personality can be harnessed to a money-making campaign, and the other conditions that make for success in London are in many cases absent in the provinces.

There are certain principles of money-raising that are common to all charitable appeals. There is, first, the appeal by letter. Only a genius can write a circular letter that really touches the charitable vein in the public. Most letters sent out by hospitals fail, either because they are cold and impersonal or because they contain lengthy "sob-stuff." If the letter method is adopted, it will be found that it is worth spending money and time on it. A badly printed letter with an obviously faked signature from a rubber stamp, and half-penny postage, is simply asking to be put into the waste-paper basket. To be effective a letter must have all the signs of being personal. The name and address of the prospective contributor should be carefully filled in, in order that the letter may look like a specially typewritten one and not a circular. The signature should be written, if not by the actual person whose name is used, at least by a proxy. It is sometimes worth while to use an odd-shaped envelope, in order that this particular letter may be conspicuous and attract attention. There are two kinds of letter which have what is known in the commercial world as "pulling power." The one is a general appeal giving facts; this usually produces a few substantial cheques from wealthier men. The other, giving some concrete story, with a romantic touch if possible, of the way in which the hospital is helping, may well bring in a flood of small contributions. It must be recognised that thousands of pounds are wasted annually on tons of charitable appeals that produce no result whatsoever.

Undoubtedly, in charitable work as in business, the personal appeal pays. Shoe-leather is what counts. A man will give to a personal caller at his house, who is able to explain the needs of the local hospital, far more readily than he will respond to appeals from the hoardings and the platform or by letter. For this reason committees of men and women who are prepared to solicit contributions, provided that they have the right personalities, are the most effective means of attaining success, but they must not shirk their work by asking for subscriptions over the telephone, as has sometimes been done. The magnetism of personality is then lost, and many a person is ready to refuse, possibly rudely, to some unseen beggar at the other end of a telephone what he would give gladly after hearing the case from a visible caller.

It is questionable whether the personal call made by the paid canvasser is really effective. There is a suspicion abroad that commissions have been too large. The voluntary worker who can make it clear that he is asking a friend or neighbour to subscribe because he has the welfare of the local hospital at heart, and not because he wishes to make a percentage of the contribution for himself, is the man most likely to be successful. If one or two canvassers are employed on a commission basis, this should be plainly stated, for the man in the street likes plain truth and realises that raising money costs money, but objects to the possibility of secret commissions.

There are many advocates of the poster. In London there has been a definite appeal to the emotions from the hoardings. Some students of the subject are inclined to think that this is largely wasted money. Other publicity experts declare that it "creates atmosphere." It appears to the writer that the emotional appeals during the war by recruiting and other posters have toughened the heartstrings of the public. There has been so much exaggeration that the average man in the street is not as much affected by the poster as he was in pre-war days. There is, in fact, far more need to-day in presenting the case of a hospital, both on a poster and in a pamphlet, to base the appeal on facts rather than on imaginative emotionalism, and even the facts should be handled with some restraint.

Another method, which has fortunately been abandoned by many of the more far-seeing hospitals, is that of organising some sort of entertainment and sending out tickets to possible subscribers, asking them either to return the tickets or to send a cheque. There is an importunity about this method that annoys the normal man, who makes a rule of throwing such literature into the fire unless he is specially interested in the object of the charity.

A rule that pays in America and will in time have to be adopted here is that of limiting the expenses of all entertainments given for charitable objects. There have been dances given in London within the last two years out of which only the professional organisers have benefited. A rule made by one social organisation is that no entertainment for raising money shall be organised if the expenses are likely to exceed 20 per cent. of the gross proceeds. The public is becoming rather wary of charitable balls, fêtes and entertainments designed to extract money painlessly. Where this method is adopted during the present hard times, when both the nation and the individual have to consider economy, it is important that it should be known that the expenses will be as low as possible.

Finally, many a hospital fails to follow up: a contributor gives his guinea to a personal caller, but after that little or nothing is done to foster his interest and make him feel a part of the institution. It is a good rule in business to follow up customers, and it is one that might well be carried out by all those responsible for hospital finance.

It is not pretended that the suggestions given above are new or comprehensive. They represent certain common-sense and rather obvious rules that are sometimes overlooked. The guiding rules, however, in charitable money-raising as in commerce, are that the plan of campaign should be carefully thought out beforehand, that the psychology of those to whom the appeals are to be sent should be considered, and economy of effort achieved.

